# Emerging and evolving values in the changing landscape of genomics

**DOI:** 10.3389/fgene.2025.1566291

**Published:** 2025-04-25

**Authors:** Maria Siermann, Riya Mohan, Eline M. Bunnik, Anne Cambon-Thomsen, Ruth Chadwick, Martina C. Cornel, Johannes J. M. van Delden, Yann Joly, Fruzsina Molnár-Gábor, Maria Pilar Nicolás Jiménez, Wim Pinxten, Emmanuelle Rial-Sebbag, Mahsa Shabani, Eva Van Steijvoort, Susan E. Wallace, Ma’n H. Zawati, Bartha Maria Knoppers, Pascal Borry

**Affiliations:** ^1^ Centre for Biomedical Ethics and Law, Department of Public Health and Primary Care, KU Leuven, Leuven, Belgium; ^2^ Department of Health, Ethics, and Society, GROW Research Institute for Oncology and Reproduction, Maastricht University, Maastricht, Netherlands; ^3^ Section Medical Ethics, Philosophy and History of Medicine, Department of Public Health, Erasmus MC, University Medical Centre Rotterdam, Rotterdam, Netherlands; ^4^ Centre d’épidémiologie et de recherche en santé des populations (CERPOP), UMR 1295, BIOETHICS Team, Institut national de la santé et de la recherche médicale (INSERM), University of Toulouse, Toulouse, France; ^5^ Centre national de la recherche scientifique (CNRS), Toulouse, France; ^6^ School of Social Sciences, Cardiff University, Cardiff, United Kingdom; ^7^ Department of Human Genetics, Amsterdam UMC, Vrije Universiteit Amsterdam, Amsterdam, Netherlands; ^8^ Amsterdam Public Health Research Institute, Amsterdam, Netherlands; ^9^ Julius Center for Health Sciences and Primary Care, University Medical Centre Utrecht, Utrecht, Netherlands; ^10^ Department of Human Genetics, Centre of Genomics and Policy, Victor Phillip Dahdaleh Institute of Genomic Medicine, McGill University, Montreal, Canada; ^11^ BioQuant Center and Faculty of Law, Heidelberg University, Heidelberg, Germany; ^12^ Faculty of Law, Chair in Law and the Human Genome Net, University of the Basque Country, Bilbao, Spain; ^13^ Healthcare and Ethics Group, Faculty of Medicine and Life Sciences, Hasselt University, Hasselt, Belgium; ^14^ Law Centre for Health and Life, Faculty of Law, University of Amsterdam, Amsterdam, Netherlands; ^15^ Department of Health Sciences, University of Leicester, Leicester, United Kingdom

**Keywords:** genetics, genomics, ethics, ELSI, equity, collective responsibility, sustainability

## Abstract

Recent advances in human genomics have transformed the field, leading to increased integration of genomics into mainstream clinical care, broadening the potential of personalized medicine, and expanding data generation and sharing. From the outset, genetics and genomics have given rise to a broad array of ethical concerns, including issues related to discrimination and stigmatization, informed consent, and reporting requirements of secondary findings. Ethics considerations and trends have evolved in parallel with the rapid technological progress in genomics. Like other transformative technologies, genomic innovations are governed by a combination of laws and ethics guidelines to ensure their responsible implementation. In this manuscript, we propose three key values that are crucial and timely to address now: equity, collective responsibility in the mainstreaming of genomics, and, sustainability. Equity warrants renewed attention due to its critical role in ensuring fair access to genomic innovations and promoting equality within society at large. Collective responsibility in the mainstreaming of genomics is equally important, especially as genomics becomes more broadly available in healthcare and to the broader public, thereby emphasizing shared accountability in its ethical application. Finally, in a context of scarcity of financial, personnel and environmental resources, sustainability needs to be considered to ensure the future of responsible governance in research and healthcare. The goal is to ensure equal access to genomic innovations, promote the ethically responsible use of genomic technologies, and support the long-term sustainability of the field.

## Introduction

Over the past 3 decades, the field of human genomics has advanced exponentially. The growing availability and long-term storage of large genomic datasets, alongside significant improvements in sequencing technology and computational genomics, have enabled researchers and clinicians to uncover previously unknown parts of the human genome, gain a deeper understanding of causes of disease, and identify factors influencing gene expression (All of Us Research Program Genomics, 2024). These developments have also expanded the availability of genetic testing possibilities and tools, facilitated more detailed data analyses, and increased the potential for the use of genomics in diagnosis and prevention of disease (Wang et al., 2022). Additionally, genomics is becoming increasingly integrated into healthcare and population screening (Foss et al., 2022; Tozzo et al., 2023).

Advances in genomics have not only improved diagnostic tools in clinical settings but also expanded other fields, such as pharmacogenomics. This includes novel treatments, such as cell and gene therapies, which address the underlying genetic causes of some rare and severe diseases rather than merely managing symptoms (Adashi et al., 2024). Currently, there are more than fifteen such therapies approved by the European Medicines Agency, targeting conditions ranging from rare eye conditions to Spinal Muscular Atrophy (SMA) (Paul-Ehrlich-Institut, 2024). This growth in gene therapies as well as other innovative clinical genomic applications has significantly advanced the field of personalized medicine. Personalized medicine entails using an individual’s genome to personalize therapeutic strategies, disease prevention and identification of predisposition or conditions to their specific needs in a timely and targeted matter. For instance, a better understanding of patients’ drug-genome interactions could potentially help patients avoid adverse drug reactions and contribute to more effective prescription, though drug-gene interactions are very complex and adverse reactions cannot be fully eliminated (Pirmohamed, 2023). However, as we will highlight, significant scientific and ethical challenges remain.

Ethical considerations and trends have evolved in parallel with the rapid technological progress in genomics. Like other transformative technologies, genomic innovations are governed by a combination of laws and ethics guidelines to ensure their responsible use (Johnson et al., 2020). One of the first ethics frameworks for genomics was outlined in 1994 by Knoppers and Chadwick after analyzing and synthesizing broad international opinions on the Human Genome Project and the broader genomics field. Their work established five core ethical values relevant to genomics: autonomy, privacy, justice, equity, and quality (out of respect for human dignity). These values were intended to establish areas of international ethical consensus that could provide future direction for guidance and regulation of genomics (Knoppers and Chadwick, 1994). By 2005, new trends in ethics of genomics were elucidated, based on greater understanding of the complexity of genomics, its potential psychological and socio-economic impact, and the increasing emphasis on public involvement in policy and ethics: reciprocity, mutuality, solidarity, citizenry, and universality (Knoppers and Chadwick, 2005). A decade later, as genomics was becoming increasingly globalized, Knoppers and Chadwick identified six additional ethics and policy considerations: governance, security, empowerment, transparency, the right not to know, and globalization (Knoppers and Chadwick, 2015).

These trends reflect the developments at specific moments in time. While many of these remain relevant to different degrees, new developments in genomics and society, marked by increased sharing of genomic information and its integration into healthcare at large, ask for careful attention to topical ethics issues. We therefore propose three key values that are especially crucial and timely to address now: equity, collective responsibility in the mainstreaming of genomics, and sustainability. Equity is a value that warrants renewed attention due to its critical role in ensuring fair access to genomic innovations and promoting equality within society at large. Collective responsibility in the mainstreaming of genomics is equally important, especially as genomics becomes more broadly available in healthcare and to the broader public, emphasizing shared accountability in its ethical application. Finally, in a context of scarcity of financial, personnel and environmental resources, sustainability needs to be considered to ensure the future of responsible governance in research and healthcare. The selection of these values is based on synthesis and workshop discussion of key issues related to genomics in the current age as suggested by the experts involved in this paper. Analysis of recent literature also demonstrates the importance of these values for agenda-setting for a responsible future of genomics. As the values of the past remain relevant and have been crucial in influencing the current ecosystem for genomics, we emphasize the connections between our three key values and the values outlined earlier by Knoppers and Chadwick throughout this article. Through the values proposed in this paper, we aim to help diverse stakeholders, including clinicians, researchers, regulators, healthcare administrators, ethics bodies (such as research ethics committees and ethicists) and industry members, shape an ethical, inclusive, and innovative future for genomics. Each section will therefore conclude with a series of practical steps for the possible future translation of the three emerging values.

## Equity

Equity, one of the original five values outlined by Knoppers and Chadwick in 1994, remains a cornerstone in the evolving field of genomics and health to this day. Despite significant changes in the genomics landscape over the past 3 decades, the importance of equity continues. Equity in genomics includes the fair representation of diverse populations in research and the equitable distribution of benefits from scientific advances. This means ensuring that diverse groups are not only included in genomic studies but also that their needs are considered and their rights are respected, particularly their right to both contribute to and benefit from scientific discoveries (Yotova and Knoppers, 2020). In that way it also links to the value of reciprocity and exchange within research, as proposed by Knoppers and Chadwick (2005). At present, most genomic data sets are dominated by data from populations of European ancestry, which creates gaps in the representation of individuals from other parts of the world. As a result, the validity of healthcare solutions may be compromised for individuals from these underrepresented groups, potentially harming the universality as well as the quality of genomics research and care (Knoppers and Chadwick, 2005; Knoppers and Chadwick, 1994).

This underrepresentation of certain groups in genomic data sets limits the effectiveness and accessibility of genomic healthcare, including genetic screening and testing, pharmacogenomics and precision medicine interventions (National Academies of Sciences Engineering and Medicine, 2018; Shaaban and Ji, 2023). While progress is being made to include more diverse populations in research and ensure equitable benefit-sharing, unequal representation persists in genomic research. To prevent exacerbating health disparities, genomic databases should be further improved through diversification (Bentley et al., 2017; Madden et al., 2024). Additionally, it is essential to collaborate with communities and to pay attention to the broader socio-political and historical contexts, which ensures that research and healthcare technologies are tailored to meet the needs and values of the diverse groups they should be serving (Hardcastle et al., 2024), thereby addressing citizenry and justice concerns (Knoppers and Chadwick, 2005; Knoppers and Chadwick, 1994).

It is also important to consider the potential of genomic data to (consciously or unconsciously) advance narratives that hinder equity. Geographic genomic ancestry categories can be mistaken with race or ethnicity categories, leading to the false assumptions that there are inherent biological differences between race or ethnicity categories (Fuentes et al., 2019; Duello et al., 2021). Some researchers search for racial differences, referred to as “race science,” which is historically linked to eugenic practices (Newman and Georgiou, 2024; BSGM and ESHG, 2024). Although it is well-documented and widely recognized in academic fields that race is a social construct and not rooted in biological differences, this distinction may not be fully understood by the general public (Braveman and Parker Dominguez, 2021). The (mis)use of such ancestry categories in this way can lead to the misrepresentation of human diversity and unjustly provide “scientific justification” for discriminatory beliefs (Blell and Hunter, 2019).

Addressing equity, furthermore, includes ensuring that genomic healthcare is accessible to all individuals and groups, regardless of financial or geographic barriers. To date, genomic testing and counselling services are not equally accessible, both within and between countries, due to factors such as cost, geographic location, and differing levels of knowledge (Best et al., 2022; Khoury et al., 2022). While developments in personalized medicine have the potential to reduce inequities by tailoring care to an individual’s specific health needs and (genomic) information (Madden et al., 2024; Ory et al., 2023), it could also widen equity gaps due to its high costs and resource demands (Green et al., 2023). Thus, it is crucial that personalized care is not reserved only for those who can afford it.

Additionally, some conditions receive more attention than others regarding therapy developments, highlighting inequities in the research agenda itself. Furthermore, new genomic technologies, such as gene editing interventions, are becoming increasingly inaccessible due to their cost and limited availability and are often restricted to high-income countries. For instance, Casgevy™, the first CRISPR-based gene therapy approved by the FDA and EMA for treating sickle-cell disease, costs millions of US dollars per dose, which makes it inaccessible to most individuals who could benefit from it. Furthermore, it is not available in many of the countries where sickle-cell disease is most prevalent (Sheridan, 2024). This high price tag also applies to other gene therapies, such as for hemophilia and SMA (Wong et al., 2023). It is thus important to determine whether structural measures can be taken to improve equity within genomics, emphasizing the need for governance of such developments (Knoppers and Chadwick, 2015). For instance, licensing or sharing patents for these technologies with low- and middle-income countries, or fostering public-private partnerships between hospitals, pharmaceutical manufacturers and regulators to negotiate the prices of otherwise costly patented innovations, could increase global access to their health benefits (National Academies of Sciences Engineering and Medicine, 2018; Moreno et al., 2019). However, it is essential to implement these strategies with safeguards to prevent conflicts of interest and ensure fairness in their execution.

**Practical steps to consider: equity**

• Addressing equity and diversity when establishing and using genomic databases.
• Prioritizing inclusion of underrepresented populations in funding for genomic research.
• Paying careful attention to the correct use of ancestry categories.
• Condemning practices of ‘race science’ and calling out its flawed nature and discriminatory consequences.
• Considering equity in the development of genomic healthcare programs and paying attention to how to reach those who are harder to include for geographical, knowledge and/or financial reasons.

## Collective responsibility in the mainstreaming of genomics

Genomic screening and genome-wide analysis are unlocking unprecedented amounts of health information. This shift toward data-driven approaches has facilitated the integration of genomics into healthcare and public health screening efforts, positioning genomic data as a key component of modern healthcare (McNeill, 2022; Mighton et al., 2022). This mainstreaming also means that a broader range of stakeholders (including various healthcare professionals, patients, ethics bodies, regulators, and industry professionals) will be involved in genomic screening and testing, which raises important questions about how to responsibly integrate genomic testing into healthcare systems (Rahman and Barwell, 2024; White et al., 2020). Rigorous assessment of the validity and utility of genomic applications prior to their introduction and widespread adoption is vital in that regard (Milko and Khoury, 2022), linking back to the value of scientific quality in order to respect human dignity (Knoppers and Chadwick, 1994).

As genomics is increasingly becoming an aspect of clinical care and public health screening (Alarcon Garavito et al., 2023), the importance of genomic literacy and informed consent becomes increasingly relevant for the responsible development and clinical integration of new tools and technologies (Bunnik et al., 2021; Cormack et al., 2024). While the COVID-19 pandemic may have contributed to a broader public understanding of genomics, recent years have also brought a rise in misinformation and conspiracy theories surrounding science (van der Linden, 2022). Adequate understanding is necessary for obtaining valid informed consent. The increasing amount and complexity of genetic information one can obtain (especially probabilistic information, such as polygenic risk scores) could potentially complicate ensuring fully informed consent by leading to confusion or information overload for both patients and healthcare professionals (Siermann et al., 2024b; Andreoli et al., 2024; Bunnik et al., 2021). It also bears consideration that more diverse stakeholders (including non-specialist healthcare professionals, policymakers, manufacturers, citizens, ethics bodies) will be involved in genomics in the future due to its mainstreaming. While it should not be assumed that some individuals will lack understanding based on their background, it is crucial that all stakeholders involved in genomics develop an adequate understanding of genomics, encompassing its scientific, policy and ethical dimensions (Siermann et al., 2024a; Vos et al., 2017). This collective responsibility for improving genomic literacy, such as via training, education and awareness raising in society at large, is necessary for the accountable ordering, reporting, and decision-making of matters of genomic testing and screening. If a stakeholder lacks adequate knowledge, they should be able to easily access relevant information from an expert. Supporting interdisciplinarity within genomics is therefore important.

Collective responsibility in genomics not only requires improving genomic literacy but also ensuring that individuals can make informed and autonomous choices, in line with the values of autonomy and empowerment (Knoppers and Chadwick, 1994; Knoppers and Chadwick, 2015), especially in the context of population screening. While participation in genomic screening programs can be stimulated for health benefits, it is vital that individuals do not experience undue and inordinate pressure to participate by healthcare professionals, family members, commercial offerings, or societal expectations. If screening options, such as non-invasive prenatal screening, become more routinized, it could lead users to feel obligated to participate, driven by a perceived responsibility as citizens or (prospective) parents (Schone-Seifert and Junker, 2021; Kater-Kuipers et al., 2018; Garcia et al., 2022). Attention should thus always be paid to counselling and informing individuals in ways that make actual informed-decision making about genomics possible.

Individuals, healthcare professionals, and researchers must be careful with the so-called “technological imperative,” where a tool is used simply because it exists rather than because it is needed. Instead, the focus should be on critical evaluation of screening programs before adoption, and on facilitating informed decision-making about technology utilization (Laberge and Burke, 2017). In discussing informed consent, important considerations arise about the type of information that should be reported in individual and population genetic screenings, particularly regarding secondary findings (Christenhusz et al., 2013; de Wert and Dondorp, 2019; Saelaert et al., 2018; Vears and Amor, 2022; Bunnik et al., 2011). To facilitate well-informed and autonomous choices about genetic testing, it is important to understand what information people wish to receive [respecting the individual’s right not to know (Knoppers and Chadwick, 2015)], and how they prefer it to be presented. Additionally, the value of mutuality and question of sharing genomic information with family members needs to be part of discussions in genomic mainstreaming, as genomic information can have implications beyond the individual (Knoppers and Chadwick, 2005). Finally, the treatment of genomic information as inherently unique or distinct from other medical data (i.e., genetic exceptionalism) is to be avoided to realize responsible integration of genomics in healthcare at large (Mcnally et al., 2004).

**Practical steps to consider: collective responsibility**

• Establishing regulations for continuous monitoring of new genomic technologies and applications with comprehensive assessment of the validity and utility of these tools before and after widespread adoption.
• Improving genomic literacy via educational programs for healthcare professionals, medical students, policymakers, and members of the public.
• Developing guidelines for informed decision-making in genomic testing and screening that consider and clarify the complexities of genomics.

## Sustainability

Sustainability should be a crucial consideration for the present and future of genomics, particularly as globalization and big data increasingly integrate genomics into society and healthcare systems. Sustainability encompasses multiple dimensions: operational, social, financial, and environmental. One key question for operational sustainability is whether genomic data, research, and innovations are being used in ways that can be maintained by existing infrastructures. This highlights the value of governance (Knoppers and Chadwick, 2015) and the importance of integrating foresight and flexibility in the governance of genomics to ensure health systems can adapt to growing and evolving demands. This also relates to the globalization and universality of genomics (Knoppers and Chadwick, 2005; Knoppers and Chadwick, 2015). For instance, infrastructure promoting harmonization, data interoperability, and data visitation strategies are important because they facilitate the exchange of knowledge and enable cross-cultural learning across different countries and regions (Pang, 2002). Furthermore, greater consideration needs to be given to including low- and middle-income countries, which often have limited financial and technological capacity, in the global sharing of genomic knowledge and tools. Responsible use of the FAIR (findability, accessibility, interoperability, reusability) principles, is also relevant here (Boeckhout et al., 2018). Ensuring equitable global access is vital for fostering a truly inclusive and sustainable operational future for genomics, emphasizing the need for solidarity (Knoppers and Chadwick, 2005).

The social sustainability of genomics must be accounted for as well, particularly concerning data sharing. Though the sharing of genomic data offers significant benefits for science and public health (Hulsen et al., 2019), it is important to evaluate how and where that data is used, to uphold the privacy and safety of genomic data in a global context, adhering to the FAIR principles (Boeckhout et al., 2018). It is critical that, alongside calls for open science and sharing of data, individuals voluntarily consent to provide their data and are fully informed of the implications. This new era of genomics calls for updated governance frameworks that appropriately consider privacy protection, security, confidentiality and consent (Mostert et al., 2016). Ideally, stakeholder involvement or co-creation should be included into policy development to ensure governance meets the long-term needs of various actors (Okun et al., 2023; Lemke and Harris-Wai, 2015; Nunn et al., 2019). For instance, building trust, fostering social license, capacity building, fair benefit sharing, co-creation, and implementing quality data management practices could, in some contexts, support the development of socially sustainable genomics (Muller et al., 2021). Social sustainability of genomics thereby builds upon values such as privacy, security, autonomy, governance, citizenry, quality and transparency (Knoppers and Chadwick, 2005; Knoppers and Chadwick, 2015; Knoppers and Chadwick, 1994).

The financial sustainability of genomics is also important, as genomics research and innovations, such as new tests and therapies, often entail significant upfront costs. However, these genomic technologies also have the potential to address challenges of scarcity and inefficiencies in healthcare by personalizing clinical care and by streamlining processes and delivering various public health benefits (Molster et al., 2018; Roberts et al., 2014). To maximize the public health potential of genomics, equity of access must be prioritized and balanced with social and operational sustainability considerations. Large-scale genetic and data-sharing initiatives underscore the need to balance public and individual interests, respecting the values of solidarity as well as transparency (Knoppers and Chadwick, 2005; Knoppers and Chadwick, 2015). For example, if hospitals collect data for primary purposes, which is later used for secondary research, efforts should be made to align (sometimes overlapping) public and private interests (Prainsack et al., 2022). Developing a sustainable and transparent approach in data ecosystems requires reducing power asymmetries, defining clear conditions for commercial involvement, and implementing fair benefit-sharing mechanisms. These measures are essential to ensure equitable and ethical secondary use of genomic data while fostering trust and sustainability (Cervera de la Cruz and Shabani, 2023; Berkman et al., 2016).

Finally, sustainability must be considered from an environmental perspective. This builds upon values such as globalization, solidarity and universality, acknowledging the global nature and consequences of genomics developments (Knoppers and Chadwick, 2005; Knoppers and Chadwick, 2015). Innovations in genomics, such as the sequencing and analysis of large genetic datasets, can have significant environmental impacts due to the energy demands of bioinformatics processes sequencing (Grealey et al., 2022). Steps should be taken to reduce the carbon footprint of these energy-intensive activities. Furthermore, the increasing integration of artificial intelligence, automation, and digital healthcare into genomics could further exacerbate its environmental impact, given the substantial energy requirements of AI technologies (Tamburrini, 2022). To address these environmental challenges, it is essential to incorporate considerations of sustainability into decision-making, policymaking, and further research (Gibney, 2022). Additionally, understanding the interplay between environmental factors and genomics could enhance our understanding of human genomics and disease development while promoting a broader and more environmentally conscious approach to genomics research and applications.

**Practical steps to consider: sustainability**

• Considering the long-term operational, social, financial, and environmental sustainability of genomics, for which inclusion of low- and middle-income countries is crucial.
• Ensuring governance frameworks remain dynamic and anticipatory considering the changing regulatory environments surrounding consent, privacy, and security requirements.
• Improving the transparency of data usage in private and public settings and implementing benefit-sharing mechanisms for diverse populations.
• Reducing the carbon footprints of energy-intensive genomics procedures and investing in long-term environmental strategies surrounding bioinformatics processing.

## Conclusion

The field of human genomics has witnessed remarkable growth over the past decade. While there is still much to achieve in genomic research, genomics is becoming increasingly embedded in healthcare systems and more easily accessible for patients. Furthermore, technological developments have led to the possibility of generating and sharing large amounts of genomic data. These advances in genomics have been a fertile source of legislative and regulatory reforms, including enhanced data-sharing frameworks, and data privacy measures, and promoted the development of initiatives aimed at incorporating genomics into clinical care settings, preventive measures and public health programs. As the field expands, we have emphasized three key values that build upon previously recognized values: equity, collective responsibility in the mainstreaming of genomics, and sustainability. Equity is essential to ensure that all individuals can have access to, benefit from, participate in and are represented within genomic research and healthcare. Collective responsibility emphasizes the need for all stakeholders–including citizens, patients, healthcare professionals, industry experts, and policymakers–to understand the implications of genomics and make informed decisions regarding the use of genomic testing and screening. Lastly, sustainability requires that genomic research and infrastructures are designed to endure, while keeping the flexibility to evolve, with careful attention to their social and environmental impact. By prioritizing these values, the future of human genomics can promote both innovation and ethical progress, ensuring that its benefits are shared widely, equitably, and responsibly.

## Data Availability

The original contributions presented in the study are included in the article, further inquiries can be directed to the corresponding author.

## References

[B1] Adashi E. Y. Gruppuso P. A. Cohen I. G. (2024). CRISPR therapy of sickle cell disease: the dawning of the gene editing era. Am. J. Med. 137, 390–392. 10.1016/j.amjmed.2023.12.018 38184185

[B2] Alarcon Garavito G. A. Moniz T. Deom N. Redin F. Pichini A. Vindrola-Padros C. (2023). The implementation of large-scale genomic screening or diagnostic programmes: a rapid evidence review. Eur. J. Hum. Genet. 31, 282–295. 10.1038/s41431-022-01259-8 36517584 PMC9995480

[B3] All of US Research Program Genomics, I (2024). Genomic data in the all of us research program. Nature 627, 340–346. 10.1038/s41586-023-06957-x 38374255 PMC10937371

[B4] Andreoli L. Peeters H. van Steen K. Dierickx K. (2024). Taking the risk. A systematic review of ethical reasons and moral arguments in the clinical use of polygenic risk scores. Am. J. Med. Genet. A 194, e63584. 10.1002/ajmg.a.63584 38450933

[B5] Bentley A. R. Callier S. Rotimi C. N. (2017). Diversity and inclusion in genomic research: why the uneven progress? J. Community Genet. 8, 255–266. 10.1007/s12687-017-0316-6 28770442 PMC5614884

[B6] Berkman B. E. Shapiro Z. E. Eckstein L. Pike E. R. (2016). “The ethics of large-scale genomic research,” in Ethical reasoning in big data: an exploratory analysis. Editors Collmann, J. Matei S. A. (Switzerland: Springer).

[B7] Best S. Vidic N. An K. Collins F. White S. M. (2022). A systematic review of geographical inequities for accessing clinical genomic and genetic services for non-cancer related rare disease. Eur. J. Hum. Genet. 30, 645–652. 10.1038/s41431-021-01022-5 35046503 PMC9177836

[B8] Blell M. Hunter M. A. (2019). Direct-to-Consumer genetic Testing's red Herring: “genetic ancestry” and personalized medicine. Front. Med. (Lausanne) 6, 48. 10.3389/fmed.2019.00048 30984759 PMC6449432

[B9] Boeckhout M. Zielhuis G. A. Bredenoord A. L. (2018). The FAIR guiding principles for data stewardship: fair enough? Eur. J. Hum. Genet. 26, 931–936. 10.1038/s41431-018-0160-0 29777206 PMC6018669

[B10] Braveman P. Parker Dominguez T. (2021). Abandon “race.” focus on racism. Front. Public Health 9, 689462. 10.3389/fpubh.2021.689462 34557466 PMC8452910

[B11] BSGM AND ESHG (2024). BSGM & ESHG condemn attempts to resurrect discredited race science and promote the pseudoscience of eugenics. Available online at: https://www.eshg.org/news/news-details?tx_news_pi1%5Baction%5D=detail&tx_news_pi1%5Bcontroller%5D=News&tx_news_pi1%5Bnews%5D=75&cHash=0ff7cdd85b7f04cff6ca7fb0d735841e.

[B12] Bunnik E. M. Dondorp W. J. Bredenoord A. L. de Wert G. Cornel M. C. (2021). Mainstreaming informed consent for genomic sequencing: a call for action. Eur. J. Cancer 148, 405–410. 10.1016/j.ejca.2021.02.029 33784533

[B13] Bunnik E. M. Schermer M. H. N. Janssens A. C. J. W. (2011). Personal genome testing: test characteristics to clarify the discourse on ethical, legal and societal issues. BMC Med. Ethics 12, 11. 10.1186/1472-6939-12-11 21672210 PMC3141793

[B14] Cervera de la Cruz P. Shabani M. (2023). Conceptualizing fairness in the secondary use of health data for research: a scoping review. Acc. Res. 32, 233–262. 10.1080/08989621.2023.2271394 37851101

[B15] Christenhusz G. M. Devriendt K. Dierickx K. (2013). To tell or not to tell? A systematic review of ethical reflections on incidental findings arising in genetics contexts. Eur. J. Hum. Genet. 21, 248–255. 10.1038/ejhg.2012.130 22739341 PMC3573190

[B16] Cormack M. Irving K. B. Cunningham F. Fennell A. P. (2024). Mainstreaming genomic testing: pre-test counselling and informed consent. Med. J. Aust. 220, 403–406. 10.5694/mja2.52254 38479398

[B17] de Wert G. Dondorp W. (2019). Opportunistic genomic screening: ethical exploration. Clin. Genome Seq., 203–224. 10.1016/b978-0-12-813335-4.00012-x

[B18] Duello T. M. Rivedal S. Wickland C. Weller A. (2021). Race and genetics versus 'race' in genetics: a systematic review of the use of African ancestry in genetic studies. Evol. Med. Public Health 9, 232–245. 10.1093/emph/eoab018 34815885 PMC8604262

[B20] Foss K. S. O'Daniel J. M. Berg J. S. Powell S. N. Cadigan R. J. Kuczynski K. J. (2022). The rise of population genomic screening: characteristics of current programs and the need for evidence regarding Optimal implementation. J. Pers. Med. 12, 692. 10.3390/jpm12050692 35629115 PMC9145687

[B21] Fuentes A. Ackermann R. R. Athreya S. Bolnick D. Lasisi T. Lee S. H. (2019). AAPA Statement on race and racism. Am. J. Phys. Anthropol. 169, 400–402. 10.1002/ajpa.23882 31199004

[B22] Garcia E. Henneman L. Gitsels-van der Wal J. T. Martin L. Koopmanschap I. Bekker M. N. (2022). Non-invasive prenatal testing (NIPT) and pregnant women's views on good motherhood: a qualitative study. Eur. J. Hum. Genet. 30, 669–675. 10.1038/s41431-021-00945-3 34400811 PMC9177610

[B23] Gibney E. (2022). How to shrink AI’s ballooning carbon footprint. Nature 607, 648. 10.1038/d41586-022-01983-7 35854114

[B24] Grealey J. Lannelongue L. Saw W. Y. Marten J. Meric G. Ruiz-Carmona S. (2022). The carbon footprint of bioinformatics. Mol. Biol. Evol. 39, msac034. 10.1093/molbev/msac034 35143670 PMC8892942

[B25] Green S. Prainsack B. Sabatello M. (2023). Precision medicine and the problem of structural injustice. Med. Health Care Philosophy 26, 433–450. 10.1007/s11019-023-10158-8 PMC10212228 37231234

[B26] Hardcastle F. Lyle K. Horton R. Samuel G. Weller S. Ballard L. (2024). The ethical challenges of diversifying genomic data: a qualitative evidence synthesis. Camb Prism. Precis. Med. 2, e1. 10.1017/pcm.2023.20 38549845 PMC10953735

[B27] Hulsen T. Jamuar S. S. Moody A. R. Karnes J. H. Varga O. Hedensted S. (2019). From big data to precision medicine. Front. Med. (Lausanne) 6, 34. 10.3389/fmed.2019.00034 30881956 PMC6405506

[B28] Johnson S. B. Slade I. Giubilini A. Graham M. (2020). Rethinking the ethical principles of genomic medicine services. Eur. J. Hum. Genet. 28, 147–154. 10.1038/s41431-019-0507-1 31534213 PMC6974588

[B29] Kater-Kuipers A. de Beaufort I. D. Galjaard R. H. Bunnik E. M. (2018). Ethics of routine: a critical analysis of the concept of 'routinisation' in prenatal screening. J. Med. Ethics 44, 626–631. 10.1136/medethics-2017-104729 29695408

[B30] Khoury M. J. Bowen S. Dotson W. D. Drzymalla E. Green R. F. Goldstein R. (2022). Health equity in the implementation of genomics and precision medicine: a public health imperative. Genet. Med. 24, 1630–1639. 10.1016/j.gim.2022.04.009 35482015 PMC9378460

[B31] Knoppers B. M. Chadwick R. (1994). The human genome Project: under an international ethical Microscope. Science 265, 2035–2036. 10.1126/science.8091225 8091225

[B32] Knoppers B. M. Chadwick R. (2005). Human genetic research: emerging trends in ethics. Nat. Rev. Genet. 6, 75–79. 10.1038/nrg1505 15630423

[B33] Knoppers B. M. Chadwick R. (2015). The ethics weathervane. BMC Med. Ethics 16, 58. 10.1186/s12910-015-0054-4 26337535 PMC4559323

[B34] Laberge A.-M. Burke W. (2017). Avoiding the technological imperative: Criteria for genetic screening programs. OBM Genet. 01, 1. 10.21926/obm.genet.1703006

[B35] Lemke A. A. Harris-Wai J. N. (2015). Stakeholder engagement in policy development: challenges and opportunities for human genomics. Genet. Med. 17, 949–957. 10.1038/gim.2015.8 25764215 PMC4567945

[B36] Madden E. B. Hindorff L. A. Bonham V. L. Akintobi T. H. Burchard E. G. Baker K. E. (2024). Advancing genomics to improve health equity. Nat. Genet. 56, 752–757. 10.1038/s41588-024-01711-z 38684898 PMC11096049

[B37] Mcneill A. (2022). Clinical genomics testing: mainstreaming and globalising. Eur. J. Hum. Genet. 30, 747–748. 10.1038/s41431-022-01131-9 35794342 PMC9259576

[B19] Mcnally E. Cambon-thomsen A. Brazell C. Cassiman J. J. Kent A. Lindpaintner K. (2004). The 25 recommendations on the ethical, legal and social implications of genetic testing. European Commission. Luxembourg: EUR 21120 – Publications Office of the European Union.

[B38] Mighton C. Shickh S. Aguda V. Krishnapillai S. Adi-Wauran E. Bombard Y. (2022). From the patient to the population: use of genomics for population screening. Front. Genet. 13, 893832. 10.3389/fgene.2022.893832 36353115 PMC9637971

[B39] Milko L. V. Khoury M. J. (2022). Editorial: DNA-based population screening for precision public health. Front. Genet. 13, 1061329. 10.3389/fgene.2022.1061329 36353109 PMC9639452

[B40] Molster C. M. Bowman F. L. Bilkey G. A. Cho A. S. Burns B. L. Nowak K. J. (2018). The Evolution of public health genomics: exploring its past, present, and future. Front. Public Health 6, 247. 10.3389/fpubh.2018.00247 30234091 PMC6131666

[B41] Moreno P. G. Joly Y. Roskams-Edris D. (2019). Could open be the yellow brick road to innovation in genomics in North America? McGill J. Law Health 13, 117–180.

[B42] Mostert M. Bredenoord A. L. Biesaart M. C. van Delden J. J. (2016). Big Data in medical research and EU data protection law: challenges to the consent or anonymise approach. Eur. J. Hum. Genet. 24, 956–960. 10.1038/ejhg.2015.239 26554881 PMC5070890

[B43] Muller S. H. A. Kalkman S. van Thiel G. Mostert M. van Delden J. J. M. (2021). The social licence for data-intensive health research: towards co-creation, public value and trust. BMC Med. Ethics 22, 110. 10.1186/s12910-021-00677-5 34376204 PMC8353823

[B44] NATIONAL ACADEMIES OF SCIENCES ENGINEERING AND MEDICINE (2018). “Understanding disparities in access to genomic medicine: Proceedings of a workshop,” Washington (DC): National Academies Press. 30452197

[B45] Newman B. Georgiou D. (2024). Genomic research is at risk from ‘race science’ activists’ discredited ideas. Guardian. Available online at: https://www.theguardian.com/science/2024/nov/03/genomic-research-is-at-risk-from-race-science-activists-discredited-ideas.

[B46] Nunn J. S. Tiller J. Fransquet P. Lacaze P. (2019). Public involvement in global genomics research: a scoping review. Front. Public Health 7, 79. 10.3389/fpubh.2019.00079 31024880 PMC6467093

[B47] Okun S. Hanger M. Browne-James L. Montgomery T. Rafaloff G. van Delden J. J. (2023). Commitments for ethically responsible sourcing, use, and reuse of patient data in the digital age: cocreation process. J. Med. Internet Res. 25, e41095. 10.2196/41095 37145833 PMC10199386

[B48] Ory M. G. Adepoju O. E. Ramos K. S. Silva P. S. Vollmer Dahlke D. (2023). Health equity innovation in precision medicine: current challenges and future directions. Front. Public Health 11, 1119736. 10.3389/fpubh.2023.1119736 36875406 PMC9975548

[B49] Pang T. (2002). The impact of genomics on global health. Am. J. Public Health 92, 1077–1079. 10.2105/ajph.92.7.1077 12084683 PMC1447192

[B50] PAUL-EHRLICH-INSTITUT (2024). “Gene therapy medicinal products,” in Federal Gazette.

[B51] Pirmohamed M. (2023). Pharmacogenomics: current status and future perspectives. Nat. Rev. Genet. 24, 350–362. 10.1038/s41576-022-00572-8 36707729

[B52] Prainsack B. el-Sayed S. Forgo N. Szoszkiewicz L. Baumer P. (2022). Data solidarity: a blueprint for governing health futures. Lancet Digit. Health 4, e773–e774. 10.1016/S2589-7500(22)00189-3 36307191

[B53] Rahman I. Barwell J. (2024). Genomic medicine for the 21st century. Ann. R. Coll. Surg. Engl. 106, 295–299. 10.1308/rcsann.2024.0030 38555870 PMC10981982

[B54] Roberts J. S. Dolinoy D. Tarini B. (2014). Emerging issues in public health genomics. Annu. Rev. Genomics Hum. Genet. 15, 461–480. 10.1146/annurev-genom-090413-025514 25184533 PMC4229014

[B55] Saelaert M. Mertes H. de Baere E. Devisch I. (2018). Incidental or secondary findings: an integrative and patient-inclusive approach to the current debate. Eur. J. Hum. Genet. 26, 1424–1431. 10.1038/s41431-018-0200-9 29970927 PMC6138744

[B56] Schone-Seifert B. Junker C. (2021). Making use of non-invasive prenatal testing (NIPT): rethinking issues of routinization and pressure. J. Perinat. Med. 49, 959–964. 10.1515/jpm-2021-0236 34469636

[B57] Shaaban S. Ji Y. (2023). Pharmacogenomics and health disparities, are we helping? Front. Genet. 14, 1099541. 10.3389/fgene.2023.1099541 36755573 PMC9900000

[B58] Sheridan C. (2024). The world's first CRISPR therapy is approved: who will receive it? Nat. Biotechnol. 42, 3–4. 10.1038/d41587-023-00016-6 37989785

[B59] Siermann M. Phillips A. Claesen-Bengtson Z. van Steijvoort E. (2024a). Stimulating professional collective responsibility from the outset in mainstreaming genomics. J. Med. Ethics 50, 525–526. 10.1136/jme-2024-109998 38719272

[B60] Siermann M. Vermeesch J. R. Raivio T. Tsuiko O. Borry P. (2024b). Polygenic embryo screening: *quo vadis*? J. Assist. Reprod. Genet. 41, 1719–1726. 10.1007/s10815-024-03169-8 38879662 PMC11263429

[B61] Tamburrini G. (2022). The AI carbon footprint and Responsibilities of AI Scientists. Philosophies 7, 4. 10.3390/philosophies7010004

[B62] Tozzo P. Delicati A. Marcante B. Caenazzo L. (2023). Digital Biobanking and big data as a new research tool: a position paper. Healthc. (Basel) 11, 1825. 10.3390/healthcare11131825 PMC10340708 37444659

[B63] van der Linden S. (2022). Misinformation: susceptibility, spread, and interventions to immunize the public. Nat. Med. 28, 460–467. 10.1038/s41591-022-01713-6 35273402

[B64] Vears D. Amor D. J. (2022). A framework for reporting secondary and incidental findings in prenatal sequencing: when and for whom? Prenat. Diagn 42, 697–704. 10.1002/pd.6097 35032068 PMC9306573

[B65] Vos S. van Delden J. J. M. van Diest P. J. Bredenoord A. L. (2017). Moral duties of genomics researchers: why personalized medicine requires a collective approach. Trends Genet. 33, 118–128. 10.1016/j.tig.2016.11.006 28017398

[B66] Wang Y. C. Wu Y. Choi J. Allington G. Zhao S. Khanfar M. (2022). Computational genomics in the era of precision medicine: applications to variant analysis and gene therapy. J. Pers. Med. 12, 175. 10.3390/jpm12020175 35207663 PMC8878256

[B67] White S. Jacobs C. Phillips J. (2020). Mainstreaming genetics and genomics: a systematic review of the barriers and facilitators for nurses and physicians in secondary and tertiary care. Genet. Med. 22, 1149–1155. 10.1038/s41436-020-0785-6 32313152

[B68] Wong C. H. Li D. Wang N. Gruber J. Lo A. W. Conti R. M. (2023). The estimated annual financial impact of gene therapy in the United States. Gene Ther. 30, 761–773. 10.1038/s41434-023-00419-9 37935855 PMC10678302

[B69] Yotova R. Knoppers B. M. (2020). The right to benefit from science and its implications for genomic data sharing. Eur. J. Int. Law 31, 665–691. 10.1093/ejil/chaa028

